# c-Myc-PD-L1 Axis Sustained Gemcitabine-Resistance in Pancreatic Cancer

**DOI:** 10.3389/fphar.2022.851512

**Published:** 2022-05-02

**Authors:** Jingjing Yao, Min Huang, Qinghong Shen, Ming Ding, Shaofang Yu, Yajuan Guo, Yuefang Lin, Yaqiu Zheng, Wenbo Chen, Wenxin Yan, Zhongqiu Liu, Dawei Wang, Ming Hu, Linlin Lu

**Affiliations:** ^1^ Joint Laboratory for Translational Cancer Research of Chinese Medicine of the Ministry of Education of the People’s Republic of China, International Institute for Translational Chinese Medicine, Guangzhou University of Chinese Medicine, Guangzhou, China; ^2^ State Key Laboratory of Quality Research in Chinese Medicine, Macau University of Science and Technology, Macau, Macau SAR, China; ^3^ Shunde Hospital of Guangzhou University of Chinese Medicine, Guangzhou University of Chinese Medicine, Guangzhou, China; ^4^ Department of Pharmacological and Pharmaceutical Sciences, University of Houston, Houston, TX, United States

**Keywords:** gemcitabine resistance, pancreatic cancer, c-Myc, PD-L1, artesunate

## Abstract

Pancreatic cancer ranks fourth among cancer-related deaths, with a 5-years overall survival rate being below 10%. Gemcitabine (dFdC) has been considered the first-line drug for patients with pancreatic cancer. However, the clinical effectiveness is less than 20% due to drug resistance. Most importantly, overwhelming evidence suggested c-Myc and PD-L1 were generally highly expressed in pancreatic cancer patients. However, whether dFdC-resistant pancreatic cancer is associated with c-Myc and PD-L1 has not been elucidated. In our present study, we found that the expression of c-Myc and PD-L1 was markedly increased in pancreatic tumor tissues compared with adjacent tissues. Similarly, c-Myc and PD-L1 expression were also remarkably elevated in dFdC-resistant Panc-1 cells compared with parental cells. In addition, dFdC sensitivity was enhanced by the combination of dFdC and c-Myc inhibitors in Panc-1 cells. Interestingly, its sensitivity was reduced when c-Myc was overexpressed. Moreover, PD-L1 protein expression was dramatically down-regulated when treated with c-Myc inhibitors. Furthermore, artesunate (ARTS) screened from 18 compounds could reverse dFdC resistance in combination with dFdC in dFdC-resistant Panc-1 cells *in vitro* and suppressed DMBA-induced pancreatic cancer *in vivo*. In summary, our data revealed that the mechanism of dFdC resistance may be that c-Myc overexpression contributed to increased PD-L1 expression, and ARTS could overcome dFdC-resistant pancreatic cancer by inhibiting c-Myc and PD-L1. Our findings not only suggest c-Myc and PD-L1 as novel prognostic biomarkers in dFdC-resistant pancreatic cancer, but also provide ARTS as a promising candidate for overcoming dFdC resistance.

## Introduction

Pancreatic cancer is a common malignant tumor, which rarely presents symptoms before it develops to advanced stage (Kamisawa et al., 2016). Most patients have no opportunity for curative surgery at the time of diagnosis (Griffin et al., 2015). Globally, it has been reported that 458,918 new cases of pancreatic cancer were identified in 2018, resulting in 432,242 deaths, with a disappointing 5-years overall survival rate being below 10% (Rawla et al., 2019). Nearly 50% of the patients have advanced cancer and metastasis at the time of diagnosis. In this case, chemotherapy has become the treatment of choice (Sarvepalli et al., 2019). Gemcitabine (dFdC), a deoxycytidine nucleoside analog, is extensively utilized as a first-line drug for advanced pancreatic cancer. Notably, the clinical efficacy response of dFdC has been proved to be superior to its predecessor 5-fluorouracil (5-FU), which was approximately five times higher than 5-FU (23.8 *vs*. 4.8%) (Burris et al., 1997). However, the clinical effect of dFdC remains unsatisfactory due to the drug resistance in pancreatic cancer, resulting in the effectiveness being less than 20% (Binenbaum et al., 2015). Thus, the mechanism of dFdC resistance urgently requires more profound investigations.

Until now, the mechanism of dFdC resistance primarily involves the following aspects: 1) Nucleoside transporters (NT): dFdC must cross the plasma membrane *via* NT to be active. Studies have shown that the exact dFdC resistance mechanism is a decrease of human equilibrative nucleoside transporter 1 (hENT1) expression, which results in restricted intracellular uptake (Mini et al., 2006); 2) Nucleoside enzymes: Deoxycytidine kinase (dCK) is the major rate-limiting enzyme in the intracellular activation and metabolism of dFdC, and its inactivation leads to dFdC resistance (Saiki et al., 2012); 3) Tumor microenvironment: Cancer-associated fibroblasts (CAFs), the fundamental component of the tumor microenvironment, stimulate dFdC resistance and pancreatic cancer progression through the SDF-1/SATB-1 pathway (Wei et al., 2018); 4) Epithelial-mesenchymal transition (EMT): EMT could induce dFdC resistance by promoting pancreatic cancer metastasis (Zheng et al., 2015). In addition, studies have confirmed that immune escape is also one of the main mechanisms of chemoresistance (Li et al., 2020). However, whether dFdC resistance mechanisms are associated with immune escape remains unclear.

Recent studies demonstrate that programmed death ligand 1 (PD-L1) and receptor molecule PD-1 are important targets in immune checkpoint blocking therapy. PD-L1 molecules highly expressed on the tumor cells surface specifically bind to PD-1 on the T cell membrane surface to inhibit the activity and function of T cells, thereby promoting tumor immune escape and drug resistance (Zhu and Xu, 2021). PD-L1 expression in tumors is an important indicator of checkpoint immunotherapy efficacy, however, PD-L1 expression in pancreatic cancer varies from 16.7 to 90%. For example, PD-L1 surface protein expression is enhanced when stimulated with dFdC, suggesting that dFdC can alter tumor immune responses, which in turn induce tumor immune escape (Doi et al., 2017). Moreover, c-Myc is a proto-oncogene that regulates cell growth and division (Farrell and Sears, 2014). Clinical data indicate that 43.5% of pancreatic cancer patients frequently present with c-Myc overexpression and aberrant activation, which means that c-Myc is closely related to the development of pancreatic cancer (Schleger et al., 2002). Targeting c-Myc for the treatment of pancreatic cancer has been an intense focus of the cancer research community. Emerging evidence suggests that c-Myc overexpression can stimulate up-regulation of PD-L1 expression, thereby inducing immune escape (Zhou et al., 2019). However, whether dFdC resistance mechanisms is associated with c-Myc remains unknown. Currently, the reported c-Myc inhibitors are mainly 10,058-F4 and 10,074-G5, but the inhibitors have not been used in clinical treatment because they may cause serious side effects by inhibiting the proliferation of normal tissues (Soucek et al., 2008). Numerous evidences demonstrate that herbal medicine is a valuable resource for anti-tumor agents, which can reverse the chemoresistance in pancreatic cancer, thereby improving the survival and quality of life for patients. Therefore, based on the above reasons, effective natural compounds, as c-Myc inhibitors, are urgently needed to reverse dFdC resistance.

Herein, our findings revealed the effect of c-Myc and PD-L1 on dFdC resistant pancreatic cancer: 1) c-Myc and PD-L1 expression markedly raised in clinical cases of pancreatic cancer, and c-Myc expression correlated with PD-L1 expression in pancreatic cancer and may serve as prognostic predictors clinically. By constructing dFdC-resistant pancreatic cancer Panc-1 cells, our data found that the expression of c-Myc and PD-L1 was significantly elevated, suggesting c-Myc and PD-L1 may be novel prognostic biomarkers in pancreatic cancer, and inhibiting c-Myc and PD-L1 may be a potential strategy to rescue dFdC resistance in pancreatic cancer therapy. 2) By assessing the effect of natural compounds alone or in combination with dFdC in dFdC-resistant Panc-1 cells, our study revealed that ARTS in combination with dFdC could overcome dFdC resistance and inhibit DMBA-induced pancreatic cancer, implying that ARTS wound be a promising candidate to overcome dFdC resistance.

## Materials and Methods

### Chemicals and Reagents

c-Myc inhibitors (10,058-F4 and 10,074-G5) were obtained from Selleckchem (Houston, TX, USA). Artemisinin (ARTS), Dihydroartemisinin (DHA), Shikonin, Oridonin, Andrographolide, Baicalein, Sulforaphane and Luteolin (purity ≥98%) were purchased from Shanghai Yuanye Bio-Technology Co., Ltd. (Shanghai, China). RPMI-1640, Dulbecco’s Modified Eagle’s medium (DMEM), Opti-MEM and fetal bovine serum (FBS) were purchased from Gibco/BRL (Grand Island, NY, USA). Sulforhodamine B (SRB) and DMSO were purchased from Sigma-Aldrich (St. Louis, MO, USA). *Homo* c-Myc plasmid was obtained from Gene Pharma (Gene Pharma Co., Ltd., China). Primary antibodies against c-Myc and PD-L1 were purchased from Cell Signaling Technology (Beverly, MA, USA); β-actin was purchased from Santa Cruz Biotechnology (Santa Cruz, CA, USA). All secondary antibodies were purchased from Cell Signaling Technology (Beverly, MA, USA).

### Cell Culture and Transfection

Panc-1 and Mia-paca cells were obtained from Guangzhou Cellcook Biotech Co., Ltd. (Guangdong, China). All cells were culture in a 37°C, 5% CO_2_ cell incubator preserved by international institute for translational Chinese medicine, Guangzhou University of Chinese Medicine (Guangdong, China).

Panc-1 and Mia-paca cells were grown to 60–80% confluence in 6-well plates. Plasmids and Lipofectamine™ 2000 (Invitrogen) were transfected with c-Myc overexpression in Opti-MEM medium, respectively, and incubated for 20 min at room temperature. Their mixture was then added to the cells.

### Sulforhodamine B Assay

Cell viability was assessed by SRB assays. 3 × 10^3^ cells were seeded in 96-well plates (100 μL per well), and treated with different compounds for 72 h. After that, cells were fixed with 10% trichloroacetic acid and then stained with 0.057% SRB, and crystals were solubilized with 10 mM Tris-HCl. Measure the absorbance of each well at 540 nm in a microplate reader (Bio-Rad Laboratories).

### Combination Index Assay

Whether dFdC with 10,058-F4 and 10,074-G5 had synergistic, additive or antagonistic effect was analyzed by combination index (CI) method (Zhao et al., 2018). CI = CA/CxA + CB/CxB; where CxA and CxB are the concentrations of A and B alone, respectively, needed to achieve a given effect (x %). CA and CB are the concentrations of A and B needed for the same effect (x %) when the drugs are combined. The combination is considered as synergistic when the CI is < 1 and antagonistic when it is > 1, and value = 1 represents additivity.

### Induction of dFdC Resistant Panc-1 Cells

Panc-1 cells were treated with 15 nM dFdC and passaged after reaching 80% confluence. One-third of the cells continued to be maintained in culture with 15 nM dFdC and counted as R-P (passage number); one-third of the cells were tested for dFdC sensitivity; one-third were analyzed and cryopreserved.

### RT-PCR Assay

Total mRNA was extracted using Trizol reagent (Invitrogen, United States) and cDNA was synthesized by the PrimeScriptTM RT reagent Kit (TaKaRa, Shiga, Japan). PCR was performed with SYBR Green real-time PCR using an ABI 7500 system (Applied Biosystems, Foster City, CA, United States). Relative gene expression was normalized to GAPDH. All the primers were designed and obtained from Beijing Genomics Institute (Guangdong, China). The data were analyzed by 2^−ΔΔct^ method. The sequences of PCR primers MYC is: 5′-GCC​ACG​TCT​CCA​CAC​ATC​AG-3′ 5′-TCT​TGG​CAG​CAG​GAT​AGT​CCT​T-3’; sequences of PCR primers CD274 is: 5′-AAA​TGG​AAC​CTG​GCG​AAA​GC-3′ 5′-GAT​GAG​CCC​CTC​AGG​CAT​TT-3’.

### Western Blot Assay

The pancreatic cancer cells were collected and lysed on ice for 30 min, centrifuged, and quantified by the BCA protein quantification kit (Thermo Fisher, MA, USA). Then protein samples were subjected to gel electrophoresis and transferred to PVDF membranes. The proteins were incubated overnight at 4°C with primary antibodies against c-Myc, PD-L1 and β-actin diluted in 2.5% BSA, and were detected with ECL luminescent solution (Thermo Fisher Scientific).

### Animals

Male C57BL/6J mice were purchased from Guangdong Medical Laboratory Animal Center (Guangzhou, China), and maintained in the animal facility in the SPF animal laboratory (License number: SYXK (GZ) 2019–0,144) with 12 h light/dark cycle, 21 ± 2°C, humidity 50 ± 10% at International Institute for Translational Chinese Medicine, Guangzhou University of Chinese Medicine (Guangzhou, China). All the animal experiments were approved by the Animal Experiment Committee of Guangzhou University of Chinese medicine (Guangdong, China).

### DMBA-Induced Pancreatic Cancer Model

After anesthesia by pentobarbital injection into the abdominal cavity, the pancreas was exposed by laparotomy in mice, and 10 mg 7,12-dimethyl-1,2-benzanthracene (DMBA) dissolved in 0.1% trioctane triglyceride was once injected into the pancreas (Tang et al., 2021). In addition to the control group, 15 weeks later, they were randomly divided into 4 groups (*n* = 10) and then administered intragastrically with 0.9% saline, dFdC (100 mg/kg), ARTS (100 mg/kg) or dFdC combined with ARTS for 5 weeks. Ultrasound and MRI were performed to visualize pancreatic cancer development. After the end of experiment, the mice were sacrificed and the pancreatic tissues and tumors of each mouse were dissected.

### H&E Staining

Mouse pancreatic tissues were fixed in 4% paraformaldehyde, embedded in paraffin, and sectioned (5 µm). Sections were deparaffinized with xylene and rehydrated with graded ethanol. The slices were stained with hematoxylin for 5 min, then eosin for 10 s, and covered with neutral gum. The images were taken with a microscope.

### Patient Tissue Specimens

Pancreatic cancer tissue microarray (HPan-Ade120Sur-01) was obtained and analyzed from Shanghai Outdo Biotechnology Co., Ltd. There was a total of 63 cases on the tissue microarray, of which 58 cases detected paired tumor- adjacent tissue.

### Immunohistochemistry Staining

Human pancreatic tissues were fixed in 4% paraformaldehyde, embedded in paraffin, and sectioned (4 µm). Sections were deparaffinized with xylene and rehydrated, followed by antigen retrieval. The samples were incubated with anti-c-Myc and PD-L1 antibodies overnight at 4°C, followed by incubation with secondary antibodies for 1 h. Sections were stained to brownish-yellow with diaminobenzidine (DAB). The images were taken with a microscope.

### Statistical Analysis

All statistical analyses were conducted using GraphPad Prism 7.0. Significant differences were analyzed by one-way ANOVA between the samples and their respective controls. Differences were considered statistically significant at *p < 0.05, **p < 0.01 and ***p < 0.001. Survival analysis of the patients was compared by the Kaplan-Meier method.

## Results

### The Expression of c-Myc and PD-L1 was Markedly Raised in Clinical Cases of Pancreatic Cancer

**FIGURE 1:**
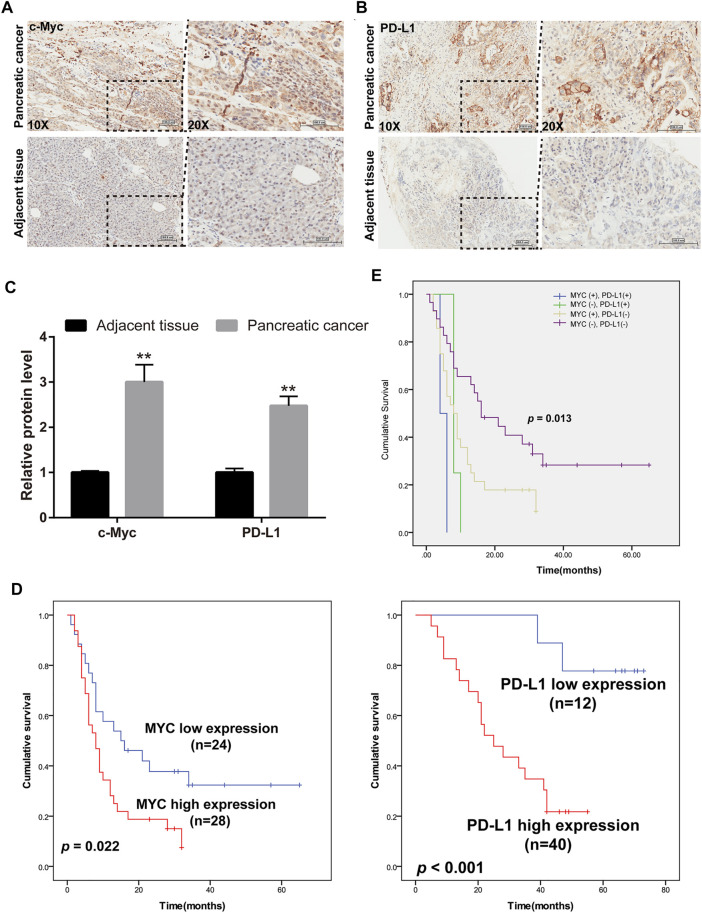
c-Myc and PD-L1 were enhanced in clinical cases of pancreatic cancer. **(A,B)** IHC analysis was used to evaluate c-Myc and PD-L1 expression in pancreatic cancer and adjacent tissues. **(C)** Composite scores of c-Myc and PD-L1 protein. **p < 0.01 *vs*. adjacent tissues. **(D)** Overall survival evaluated between pancreatic cancer patients with high and low expression of c-Myc and PD-L1. **(E)** Kaplan Meier plot of c-Myc and PD-L1. The blue curve indicates c-Myc-high and PD-L1-high (double high), the green curve indicates c-Myc-low but PD-L1-high staining, yellow shows c-Myc-high and PD-L1-low, and blue indicates double low.

Overwhelming evidence suggested that approximately 43.5% of patients with pancreatic cancer may belong to high c-Myc expression, and aberrant expression of PD-L1 predicted poor prognosis and promoted pancreatic cancer progression (Wang et al., 2020). Thus, the expression of c-Myc and PD-L1 in pancreatic cancer and adjacent tissues was conducted in HPan-Ade1 20Sur-01 microarray (Table 1 and Table 2). The results showed that the c-Myc and PD-L1 expression were markedly upregulated in pancreatic tumors compared with adjacent tissues (Figures 1A–C). The expression levels of c-Myc and PD-L1 in pancreatic cancer tissues were positively correlated at the protein level (*p* = 0.042; Table 3). Furthermore, overall survival of clinical cases of pancreatic cancer was analyzed by Kaplan-Meier, we found that patients with high c-Myc and PD-L1 expression had shorter overall survival, indicating that high c-Myc (*p* = 0.022) and PD-L1 (*p* < 0.001) expression were associated with poor prognosis of patients (Figure 1D). Moreover, we further analyzed the correlation of c-Myc/PD-L1expression with clinical outcomes by dividing 63 pancreatic cancer patients into four c-Myc/PD-L1 expression groups (c-Myc-high and PD-L1-high, c-Myc-low and PD-L1-high, c-Myc-high and PD-L1-low, c-Myc-low and PD-L1-low). The results found that PD-L1 and c-Myc double-high tumors were associated with worse overall survival compared to the other groups (*p* = 0.013; Figure 1E).

**TABLE 1:** Correlation between c-Myc expression and clinicopathological characteristics.

	Variables	Pancreatic cancer		Total	χ^2^	*p* value
		c-Myc Expression				
		Low	High			
Age (year)	≤65	20	7	27	0.088	0.766
	>65	24	7	31		
Sex	Female	16	8	24	1.891	0.169
	male	28	6	34		
Grade	I/II	25	9	34	0.244	0.621
	III	19	5	24		
T stage	T1/T2	5	3	8	0.516	0.473
	T3	27	9	36		
N stage	N0	23	9	32	0.854	0.355
	N1	19	4	23		
M stage	M0	40	14	54	1.367	0.242
	M1	4	0	4		
TNM stage	Ι/II	34	12	46	1.373	0.241
	IV	4	0	4		

**TABLE 2:** Correlation between PD-L1 expression and clinicopathological characteristics.

	Variables	Pancreatic cancer		Total	χ^2^	*p* value
		PD-L1 Expression				
		Low	High			
Age (year)	≤65	7	20	27	0.358	0.549
	>65	6	25	31		
Sex	Female	5	19	24	0.059	0.808
	male	8	26	34		
Grade	I/II	11	23	34	4.668	0.031
	III	2	22	24		
T stage	T1/T2	2	6	8	0.097	0.755
	T3	11	25	36		
N stage	N0	9	23	32	0.854	0.355
	N1	4	19	23		
M stage	M0	13	41	54	1.241	0.265
	M1	0	4	4		
TNM stage	Ι/II	13	33	46	1.528	0.216
	IV	0	4	4		

**TABLE 3:** Correlation between the expression of c-Myc and PD-L1 in pancreatic cancer.

PD-L1	c-Myc		Spearman’s Correlation		
	High	Low	Total	r	*p*-Values
High	1	4	5	0.257	0.042
Low	13	45	58	—	—
Total	14	49	63	—	—

### c-Myc and PD-L1 Were Overexpressed in dFdC-Resistant Panc-1 (Panc-1/dFdC) Cells

**FIGURE 2:**
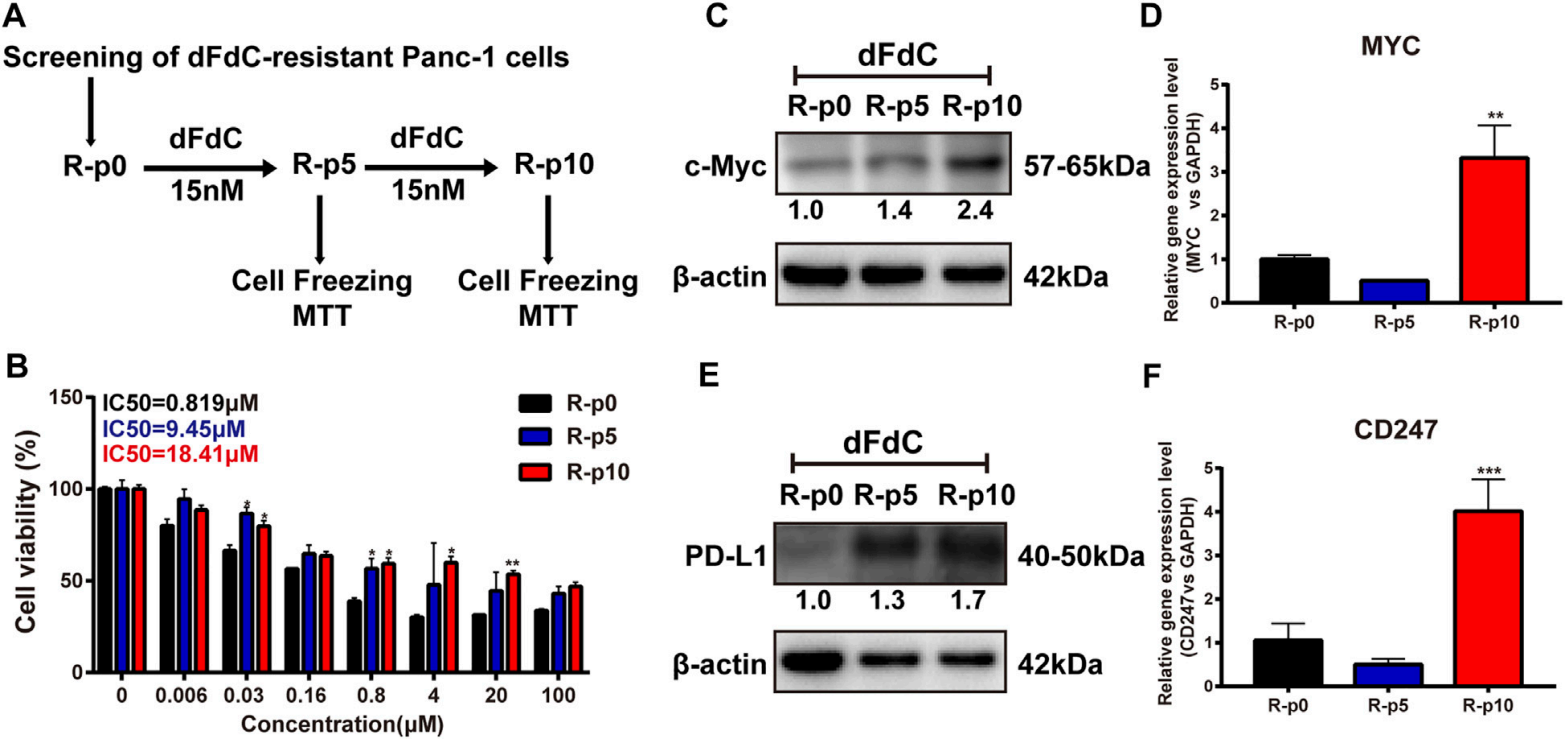
c-Myc and PD-L1 were elevated in dFdC-resistant Panc-1 (Panc-1/dFdC) cells. **(A)** Screening method of Panc-1/dFdC pancreatic cancer cells. **(B)** Panc-1/dFdC cells was established. The cell viability of the indicated cells was determined using SRB assays. **(C,E)** The protein expression of c-Myc and PD-L1 was examined in Panc-1/dFdC cells by Western blot. **(D,F)** The gene expression of MYC and CD247 was evaluated in Panc-1/dFdC cells by RT-PCR. **p < 0.01, ***p < 0.001 *vs*. without dFdC treatment (R-p0).

To comprehensively reveal whether c-Myc and PD-L1 are associated with dFdC resistance, dFdC-resistant cell lines were initially established. As showed in Figure 2A, untreated Panc-1 cells were used as parental cells (R-p0), and subsequently the cells were maintained with a low concentration of 16.7 nM dFdC at the 5th generation (R-p5) and the 10th generation (R-p10) to detect the cell proliferation of dFdC and cell cryopreservation. As the number of culture generations increased, the sensitivity of dFdC in Panc-1 cells was markedly decreased. The IC_50_ value of R-p10 enhanced by 22.5-fold compared with R-p0, indicating that the dFdC-resistant Panc-1 cell line was successfully established (Figure 2B). In addition, the gene and protein expression of c-Myc and PD-L1 in Panc-1/dFdC cells at R-p0, R-p5 and R-p10 were examined, respectively. The results suggested that the c-Myc protein and gene levels of R-p10 were significantly promoted by 2.4-fold (*p* < 0.05) and 3-fold (*p* < 0.01), respectively (Figures 2C,D). Similarly, compared with R-p0, the PD-L1 protein and gene levels of R-p10 were remarkably upregulated by 1.7-fold (*p* < 0.05) and 4-fold (*p* < 0.001), respectively (Figures 2E,F), suggesting that high c-Myc and PD-L1 expression may induce dFdC resistance.

### c-Myc Inhibitors Enhanced dFdC Sensitivity in Pancreatic Cancer Cells

**FIGURE 3:**
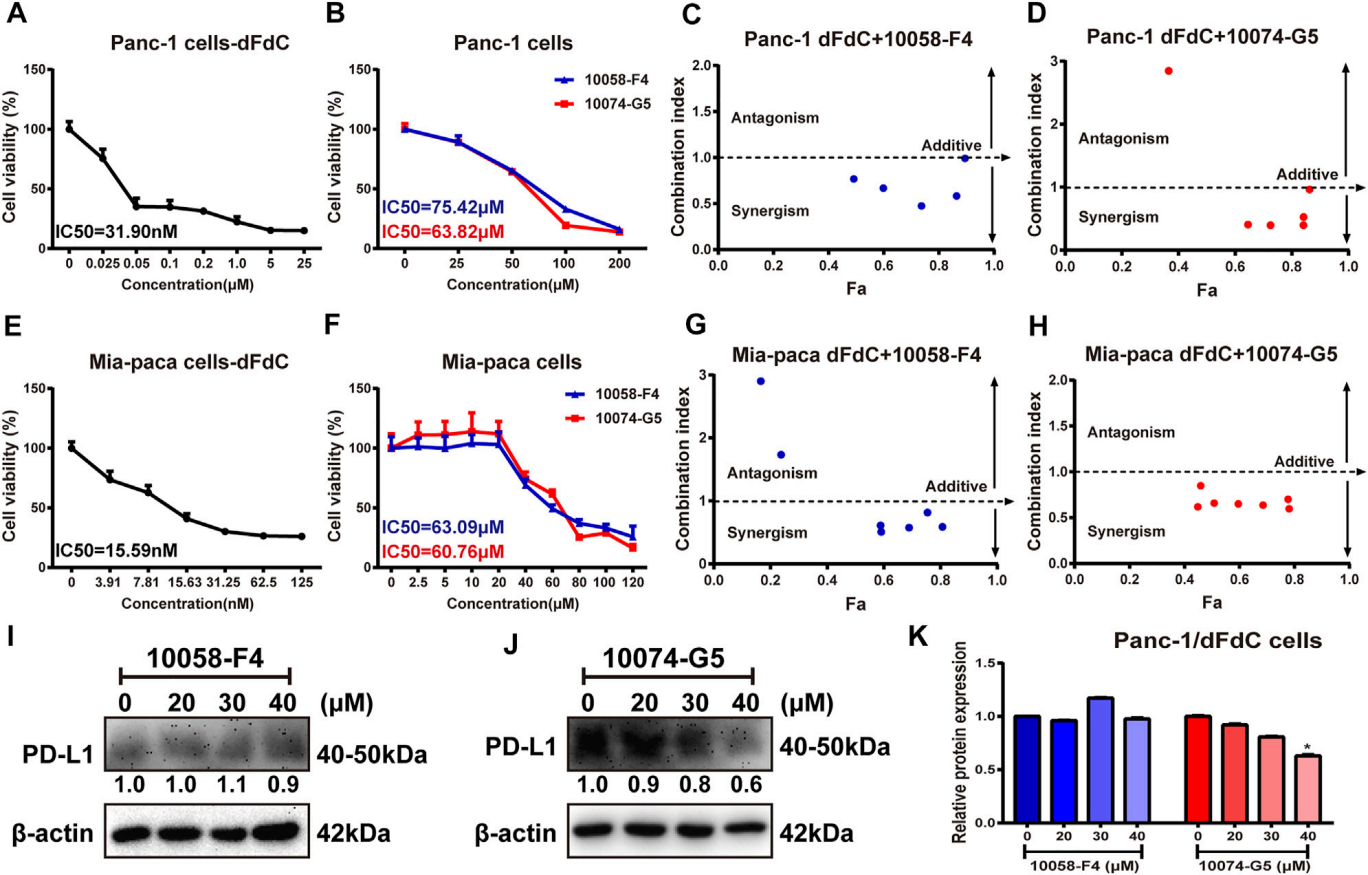
c-Myc inhibitor improved the inhibitory effect of dFdC in pancreatic cancer cells. **(A,B)** Cell viability of Panc-1 cells after dFdC and c-Myc inhibitors (10,058-F4 and 10,074-G5) treatment for 72 h (*n* = 3). **(C,D)** The synergetic effect was detected after Panc-1 cells treated with 16.7 nM dFdC (85% survival) in combination with 10,058-F4 and 10,074-G5 for 72 h. The Combination Index (CI) value was determined by CompuSyn analysis. Fa = Fraction affected. The combination is considered as synergistic when the CI is < 1 and antagonistic when it is > 1, and value = 1 represents additivity. **(E,F)** Cell viability of Mia-Paca cells after dFdC and c-Myc inhibitors (10,058-F4 and 10,074-G5) treatment for 72 h (*n* = 3). **(G,H)** The synergetic effect was detected after Mia-Paca cells treated with 2.8 nM dFdC (85% survival) in combination with 10,058-F4 and 10,074-G5 for 72 h. **(I,J)** Western blot analysis of PD-L1 expression after c-Myc inhibitors (10,058-F4 and 10,074-G5) treatment. **(K)** Quantitative Analysis of PD-L1 protein. *p < 0.05 *vs*. without c-Myc inhibitors treatment (*n* = 3).

To assess the anti-pancreatic cancer effect of dFdC and c-Myc inhibitors (10,058-F4 and 10,074-G5), we initially examined the cell viability in pancreatic cancer Panc-1 and Mia-Paca cells. When Panc-1 cells were treated by dFdC, 10,058-F4 and 10,074-G5 with different concentrations for 72 h, the IC_50_ values were 31.90 nM, 75.42 and 63.82 μM, respectively (Figures 3A,B). To further determine the synergetic role of c-Myc inhibitors and dFdC in pancreatic cancer, dFdC at 16.7 and 2.8 nM (concentration that causing 85% survival rate of Panc-1 and Mia-Paca cells, respectively) was selected for subsequent combinational experiment. At the meantime, to clearly evaluate the combination index (CI), a series of 10,058-F4 and 10,074-G5 concentrations (ranged from 5 to 240 μM) were used to treat Panc-1 and Mia-Paca cells simultaneously. As showed in Figures 3C,D, 16.7 nM of dFdC, only causing 15% of cell death by using alone, could cause 73.7 and 84.1% of cell death by combining with 60 μM of 10,058-F4 and 10,074-G5, respectively (*p* < 0.001). And the CI of dFdC with 10,058-F4 and 10,074-G5 were 0.47 and 0.39, respectively, suggesting that c-Myc inhibitor combined with dFdC improved drug sensitivity. Additionally, similar results were also observed in Mia-Paca cells. When Mia-Paca cells were treated by dFdC, 10,058-F4 and 10,074-G5 alone for 72 h, the IC_50_ values were 15.59 nM, 63.09 and 60.76 μM, respectively (Figures 3E,F). But when Mia-Paca cells were treated with dFdC combined with 10,058-F4 and 10,074-G5 simultaneously, the CI were 0.57 and 0.59 with 60 μM of 10,058-F4 and 10,074-G5, respectively (Figures 3G,H). Taken together, the above results indicated that c-Myc inhibitors (10,058-F4 and 10,074-G5) had a moderate synergistic effect with dFdC in Panc-1 and Mia-Paca cells, suggesting that c-Myc inhibitors could enhance the inhibitory effect of dFdC in pancreatic cancer cells, thereby increasing the dFdC sensitivity. Thus, Panc-1 cells were used as subsequent experiments.

Some of evidences demonstrated that PD-L1 binds to PD-1 expressed on immune T cells, thereby promoting tumor immune escape and drug resistance (Zhu and Xu, 2021). PD-L1 expression is significantly increased when treated with dFdC, which enhanced dFdC resistance (Doi et al., 2017). In agreement with previous studies (Casey et al., 2016), we found that there was no significant difference in PD-L1 expression by c-Myc inhibitor 10,058-F4 (Figures 3I,K). However, PD-L1 protein expression was dramatically down-regulated (range, 100–40%) in a dose-dependent manner, when Panc-1/dFdC cells were stimulated with different concentrations of c-Myc inhibitor 10,074-G5 (*p* < 0.05; Figures 3J,K), suggesting that c-Myc overexpression may contribute to raised PD-L1 expression.

### c-Myc Overexpression Improved dFdC Resistance in Pancreatic Cancer Cells

**FIGURE 4:**
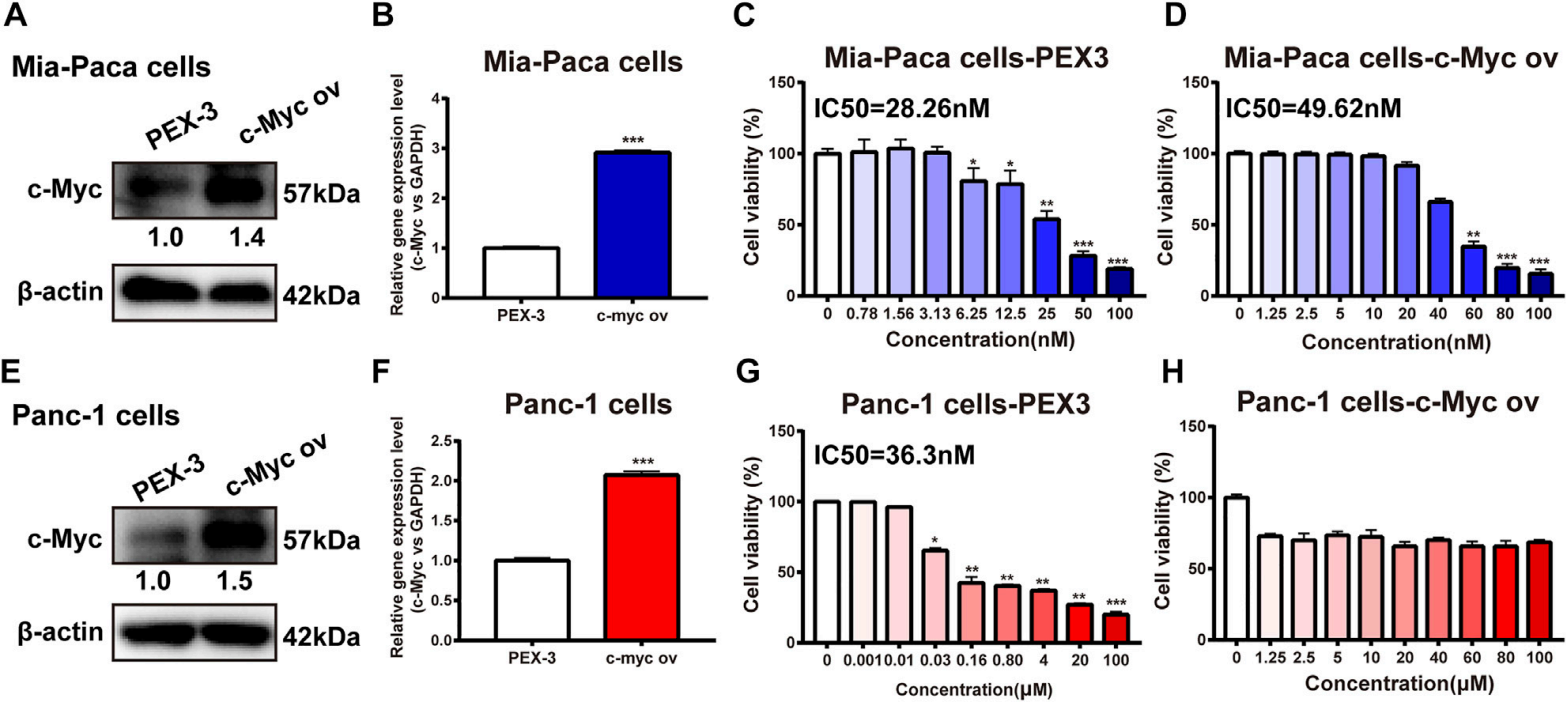
Effects of c-Myc overexpression on dFdC. **(A)** The protein expression of c-Myc was examined in empty plasmid (PEX3) and c-Myc overexpression (ov) Mia-paca cells by Western blot. **(B)** The gene expression of MYC was evaluated in empty plasmid (PEX3) and c-Myc ov Mia-paca cells by RT-PCR. ***p < 0.001 *vs*. empty plasmid (PEX3) (*n* = 3). **(C,D)** SRB assay was performed to compare cell viability against different concentrations of dFdC in PEX3 and c-Myc ov Mia-paca cells for 72 h (*n* = 3). **(E)** The protein expression of c-Myc was examined in empty plasmid (PEX3) and c-Myc ov Panc-1 cells by Western blot. **(F)** The gene expression of MYC was evaluated in empty plasmid (PEX3) and c-Myc ov Panc-1 cells by RT-PCR. ***p < 0.001 *vs*. empty plasmid (PEX3) (*n* = 3). **(G,H)** MTT assay was performed to compare cell viability against different concentrations of dFdC in PEX3 and c-Myc ov Panc-1 cells for 72 h (*n* = 3).

To further confirm the anti-pancreatic cancer effect of c-Myc overexpression on dFdC, pancreatic cancer Mia-Paca and Panc-1 cells were transfected with c-Myc overexpression plasmid. Compared with the empty plasmid (PEX3), the c-Myc protein expression was enhanced by 1.4-fold and 1.5-fold in the c-Myc over-expressed group in Mia-Paca and Panc-1cells, respectively (*p* < 0.05; Figures 4A,E). Similarly, the gene expression of c-Myc was increased by 3-fold and 2-fold, respectively (*p* < 0.001; Figures 4B,F). Moreover, the inhibitory effects of dFdC in c-Myc over-expressed Mia-Paca and Panc-1 cells were examined, respectively. Our results showed that the IC_50_ of dFdC was markedly elevated in Mia-Paca cells with overexpression of c-Myc (range, 28.26–49.62 nM; Figures 4C,D). Similarly, in the Panc-1 cell line with empty plasmid, the IC_50_ value was 36.3 nM, but when the dFdC concentration was raised to 100 μM, IC_50_ was not detected in c-Myc over-expressed cells (Figures 4G,H). Together, the results showed that the dFdC resistance on Mia-Paca and Panc-1 cells were significantly enhanced by c-Myc overexpression, and dFdC resistance in Panc-1 cells was dramatically higher than that in Mia-Paca cells.

### Artesunate Reversed dFdC Resistance in Combination With dFdC in Panc-1/dFdC Cells

**FIGURE 5:**
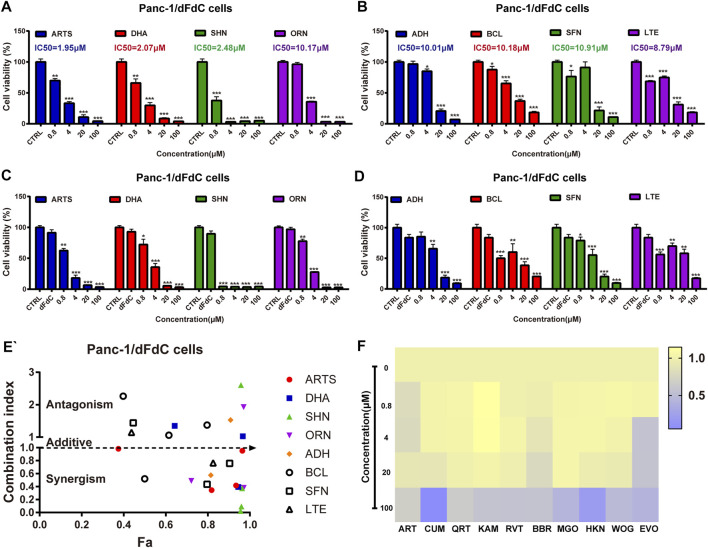
Screening of nature compounds to overcome dFdC resistance on pancreatic cancer Panc-1/dFdC cells *in vitro*. **(A,B)** SRB assay was performed to assess the cell viability of 8 compounds (Artesunate, Dihydroartemisinin, Shikonin, Oridonin, Andrographolide, Baicalein, Sulforaphane, Luteolin) for 72 h in Panc-1/dFdC cells. *p < 0.05, **p < 0.01, ***p < 0.001 *vs*. Control group (*n* = 3). **(C,D)** SRB assay was performed to assess the cell viability of 8 compounds in combination with 16.7 nM dFdC (85% survival) for 72 h in Panc-1/dFdC cells. *p < 0.05, **p < 0.01, ***p < 0.001 *vs*. Control group. **(E)** The synergetic effect was detected in Panc-1/dFdC cells treated with 16.7 nM dFdC (85% survival) in combination with compounds for 72 h. **(F)** Heat map was performed to assess the cell viability of other 10 compounds (Artemisinin, Curcumin, Quercetin, Kaempferol, Resveratrol, Berberine, Magnolol, Honokiol, Wogonin, Evodiamine) for 72 h in Panc-1/dFdC cells.

Accumulating evidence suggested that artemisinin and its derivatives suppressed dFdC-induced pancreatic cancer by down-regulating c-Myc (Wang et al., 2010). To explore whether natural compounds can be acted as c-Myc inhibitor to inhibit Panc-1/dFdC cells, 18 compounds (Artemisinin, Artesunate, Dihydroartemisinin, Shikonin, Curcumin, Oridonin, Quercetin, Kaempferol, Resveratrol, Berberine, Magnolol, Honokiol, Andrographolide, Baicalein, Sulforaphane, Luteolin, Wogonin, Evodiamine) were identified to assess their anti-proliferation effects on Panc-1/dFdC cells either alone or in combination with dFdC. The results showed that artesunate (ARTS), dihydroartemisinin (DHA), shikonin (SHN), oridonin (ORN), andrographolide (ADH), baicalein (BCL), sulforaphane (SFN) and luteolin (LTE) had remarkable inhibitory effects on Panc-1/dFdC cells in a dose-dependent manner, with IC50 of 1.95, 2.07, 2.48, 10.17, 10.01, 10.18, 10.91 and 8.79 μM, respectively (Figures 5A,B). And other compounds had little proliferative effects on Panc-1/dFdC cells (Figure 5F), indicating that ARTS could significantly inhibit dFdC-resistant Panc-1 cell growth compared with other compounds. To further determine the synergetic role of the above 8 compounds and dFdC in pancreatic cancer cells, dFdC at 16.7 nM (concentration that causing 85% survival rate of Panc-1 cells) was selected for subsequent combinational experiment. At the meantime, to clearly evaluate the CI, different concentrations of compounds (0.8–100 μM) were used to treat Panc-1/dFdC cells simultaneously. When Panc-1/dFdC cells were treated with dFdC combined with compounds simultaneously, the CI were 0.34, 1.34, 0.37, 0.48, 3.96, 2.26, 1.44 and 8.79 with 4 μM of 8 compounds, respectively. These results showed that dFdC combined with ARTS, SHN and LTE at 4 μM had synergistic effect, suggesting that ARTS had a more pronounced inhibitory effect on Panc-1/dFdC pancreatic cancer cells and may reverse dFdC resistance in combination with dFdC (Figures 5C–E).

### Artesunate in Combination With dFdC Inhibited DMBA-Induced Pancreatic Cancer in Mice

**FIGURE 6:**
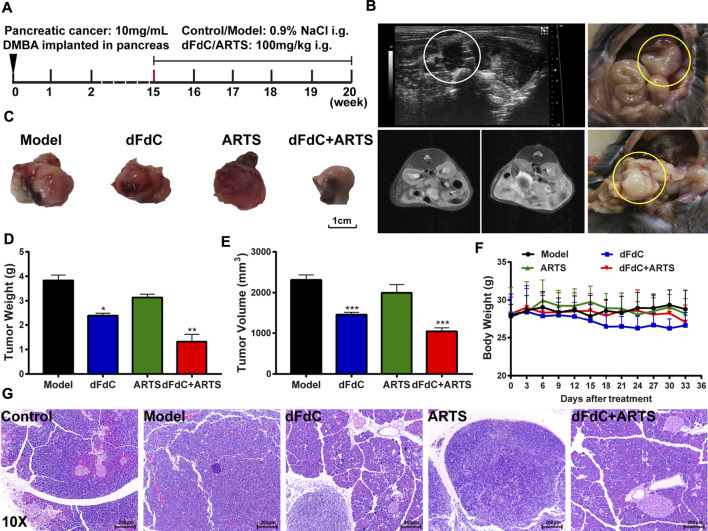
Artesunate (ARTS) in combination with dFdC inhibited DMBA-induced pancreatic cancer in mice. **(A)** The schematic diagram showed the experimental design of DMBA-induced pancreatic cancer mice. **(B)** Ultrasound and MRI analysis identified tumor formation in the pancreas. **(C)** Representative images of tumors harvested from mice each group. **(D)** The tumor weight of mice each group. **(E)** The tumor volume of mice each group. **(F)** Body weight of mice of different groups after treatments. **(G)** Representative pathological images showing the effect of ARTS combined with dFdC on pancreatic cancer. *p < 0.05, **p < 0.01, ***p < 0.001 *vs*. Model group (*n* = 3).

To analyze the effect of the combination of ARTS and dFdC on pancreatic cancer *in vivo*, pancreatic cancer model was induced with DMBA implantation in mice (Osvaldt et al., 2006). As shown in the animal protocol, 10 mg/mL (10 μL) DMBA was implanted into pancreas for 15 weeks, followed by intragastric administration of dFdC and ARTS from week 15th to week 20th (Figure 6A). Subsequently, ultrasound analysis confirmed the heterogeneous proliferation formation in mice with DMBA-induced pancreatic cancer, and pathological peritoneal effusion was observed by MRI at week 15th (Figure 6B). Moreover, we found that tumors were formed at the pancreatic site. Compared to model group, dFdC alone significantly reduced tumor weight/volume by 37.5 and 32% (*p* < 0.05), and dFdC in combination with ARTS markedly decreased tumor weight/volume by 65.5 and 50.3% (*p* < 0.01; Figures 6C–E). However, there was no significant difference in body weight in each group, indicating that dFdC has little side effect on pancreatic cancer (Figure 6F). Furthermore, HE staining showed that compared with control group, pancreatic cancer was formed in model group by significant inflammatory cell infiltration, extensive interstitial connective tissue proliferation, deeper staining of nucleus and prominent nuclear isomers in the pancreatic tissue. When dFdC and ARTS were treated alone or in combination, their pathological changes were obviously improved (Figure 6G).

## Discussion

The mechanisms by which pancreatic cancer develop resistance to chemotherapeutic agents are complex and poorly understood. Clinical studies indicated that chemoresistance is the most critical factor for limiting pancreatic cancer treatment, resulting in poor survival and poor prognosis (Wei et al., 2019). Although dFdC-based chemotherapy has improved survival in patients with pancreatic cancer, the results achieved remain limited (Binenbaum et al., 2015). Thus, identifying new targets and mechanisms to overcome dFdC resistance is a critical strategy in pancreatic cancer. In the current study, the expression abundance of c-Myc and PD-L1 was detected in clinical cases of pancreatic cancer. Our data showed that c-Myc and PD-L1 were highly expressed in pancreatic cancer, suggesting that c-Myc and PD-L1 are crucial in pancreatic cancer (Figures 1A–C). In addition, there may be a positive correlation between c-Myc and PD-L1 (Figures 1D,E; Table 3). More importantly, the survival analysis revealed that high expression of c-Myc (*p* = 0.022) and PD-L1 (*p* < 0.001) in pancreatic tumor tissues predicted a poor prognosis (Figure 1D), and PD-L1 and c-Myc double-high tumors were associated with worse overall survival compared to the other groups (*p* = 0.013; Figure 1E). Futhermore, dFdC-resistant pancreatic cancer cells were established and novel targets were identified. With the increase of cell generations, the sensitivity of dFdC in Panc-1 cells was markedly decreased. The IC_50_ value of R-p10 raised by 22.5-fold compared with R-p0 (Figure 2B). The gene and protein expression of c-Myc were higher in dFdC-resistant Panc-1 cells than the untreated Panc-1 cells (*p* < 0.05; Figures 2C,D). Similarly, PD-L1 was overexpressed in dFdC-resistant Panc-1 cells compared with the sensitive cells (*p* < 0.05; Figures 2E,F). Taken together, the above results confirmed that c-Myc and PD-L1 were involved in the resistance to dFdC in pancreatic cancer.

Mechanistically, the proto-oncogene c-Myc can regulate cytotoxicity-induced apoptosis and is commonly overexpressed in pancreatic cancer. Many studies have highlighted that c-Myc can modulate dFdC resistance in pancreatic cancer cells (Biliran et al., 2007). In line with these studies, the effects of c-Myc inhibitor and c-Myc overexpression on dFdC resistance in pancreatic cancer cells were evaluated. We observed that c-Myc inhibitors (10,058-F4 and 10,074-G5) had a moderate synergistic effect with dFdC in pancreatic cancer Panc-1 and Mia-Paca cells, suggesting that c-Myc inhibitors could enhance the growth inhibitory effect of dFdC, thereby increasing the dFdC sensitivity (Figures 3A–H). In addition, c-Myc overexpression significantly attenuated the inhibitory effect of dFdC on Mia-Paca and Panc-1 cells. The IC50 of dFdC in c-Myc-overexpressing Mia-Paca cells was significantly elevated from 28.26 to 49.62 nM, whereas the IC50 in Panc-1 cells was not detected even though the dFdC concentration increased to 100 μM (Figure 4), indicating that c-Myc overexpression could strengthen the dFdC resistance in Panc-1 cells. Interestingly, intrinsic PD-L1 can mediate dFdC resistance in pancreatic cancer (Zhang and Reyes, 2021). Overwhelming evidence revealed that c-Myc overexpression contributes to increased PD-L1 expression, thereby promoting tumor immune escape, which is also one of the major mechanisms of resistance to chemotherapeutic agents (Zhou et al., 2019). Given that c-Myc affects PD-L1 expression, thereby inducing dFdC resistance in pancreatic cancer. Consistently, our data revealed that c-Myc inhibitor 10,074-G5 dramatically decreased PD-L1 expression in a dose-dependent manner (*p* < 0.05; Figure 3J) Therefore, considering the overexpression of c-Myc and PD-L1 in pancreatic tumors, we concluded that c-Myc could trigger PD-L1, thereby may contribute to the occurrence of dFdC resistance in pancreatic cancer.

Traditional Chinese medicine (TCM) contains numerous bioactive components, some of which exert anti-pancreatic cancer effects by reversing drug resistance, providing a new drug candidate for the treatment of pancreatic cancer (Gao et al., 2021). However, whether active compounds can act as c-Myc inhibitors to reverse dFdC resistance and thus exert anti-pancreatic cancer effects remain unknown. Accumulating evidence suggests that curcumin is a phenolic compound extracted from *turmeric* that can overcome dFdC resistance in pancreatic carcinoma by inhibiting the PRC2-PVT1-c-Myc axis (Yoshida et al., 2017). Meanwhile, other evidences also indicated that oridonin is a tetracyclic diterpenoid extracted from *Camellia sinensis* that can overcome dFdC resistance in PANC-1/Gem cells by regulating LRP1/ERK/JNK signaling (Wang et al., 2019). Consistently, artemisinin (ART), a sesquiterpene lactone compound extracted from the plant *Artemisia annua*, which means it exert overwhelming anti-tumor effects against lung cancer, pancreatic cancer and breast cancer (Zyad and Tilaoui, 2018). Considering that ARTS is a safe and cost-effective natural agent, it could have enormous clinical benefits. Previous studies revealed that ARTS could inhibit human pancreatic cancer *via* a novel form of oncosis-like cell death (Du et al., 2010). However, to the best of our knowledge, it has not yet been reported whether ARTS can overcome dFdC resistance in pancreatic cancer or to inhibit Panc-1/dFdC cells as an alternative dFdC-resistant compound. In our present study, ARTS had a more pronounced inhibitory effect in Panc-1/dFdC cells (IC50 = 1.95 μM), and ARTS screened from 18 compounds combined with dFdC had remarkable synergistic effect (CI = 0.34), suggesting that ARTS may reverse dFdC resistance in combination with dFdC (Figure 5). Consistent with previous studies, ARTS in combination with dFdC inhibited DMBA-induced pancreatic cancer in mice (Figure 6), implying that ARTS could overcome dFdC resistance in pancreatic cancer by suppressing c-Myc and PD-L1 expression. Taken together, ARTS would be a promising therapeutic agent for pancreatic cancer. However, further explorations, including c-Myc and PD-L1 binding assays and clinical trials, are needed to confirm the efficacy of this compound as an adjuvant chemotherapy treatment.

## Conclusion

In conclusion, our present data revealed that c-Myc expression correlated with PD-L1 expression in pancreatic cancer and may serve as prognostic predictors clinically, indicating that restrainting c-Myc and PD-L1 overexpression in pancreatic cancer may provide a window to overcome dFdC resistance. Moreover, ARTS in combination with dFdC inhibited DMBA-induced pancreatic cancer, suggesting that ARTS may become an adjuvant dFdC-resistant agent for patients who suffer from pancreatic cancer. In addition, mechanistic studies on natural compounds such as ARTS have contributed to developing safer and more effective chemotherapeutic agents. This research provided a theoretical basis for the mechanism of dFdC resistance in pancreatic cancer, and provided potential targets and ideas for the treatment of pancreatic cancer and the exploitation of natural compounds.

## Data Availability

The original contributions presented in the study are included in the article/Supplementary Materials, further inquiries can be directed to the corresponding authors.

## Abbreviations

5-FU, 5-fluorouracil; ADH, andrographolide; ARTS, artesunate; BCL, baicalein; CI, combination index; dCK, deoxycytidine kinase; dFdC, gemcitabine; DHA, dihydroartemisinin; hENT1, human equilibrative nucleoside transporter 1; LTE, luteolin; NT, nucleoside transporters; ORN, oridonin; PD-L1, programmed death ligand 1; SFN, sulforaphane; SHN, shikonin.
